# Refractive Error Coverage and Related Sociodemographic Factors: A Study of 1.1 Million Students in Schools of Hubei Province, China

**DOI:** 10.1167/tvst.15.8.5

**Published:** 2026-08-07

**Authors:** Ouwen Duan, Lianqun Wu, Cancan Zhang, Yi Lu, Lurun Yu, Qi Gong, Qing Yuan, Lianhong Zhou

**Affiliations:** 1 Department of Ophthalmology, Renmin Hospital of Wuhan University, Wuhan, China; 2 Eye Institute and Department of Ophthalmology, Eye and ENT Hospital, Fudan University, Shanghai, China; 3 Hubei Aier Eye Hospital, Wuhan, China; 4 Department of Ophthalmology, Chongqing Hospital, Union Hospital, Tongji Medical College, Huazhong University of Science and Technology, Chongqing, China

**Keywords:** refractive error, refractive error coverage, public health, myopia

## Abstract

**Purpose:**

This study aims to investigate the prevalence of refractive error coverage and its multidimensional sociodemographic influencing factors among 1.1 million school-aged students in Hubei Province, China.

**Methods:**

This population-based, cross-sectional study (2022–2024) included 1,113,548 students selected via stratified cluster sampling across Hubei Province, China. Standardized clinical assessments comprised distance visual acuity (VA) and noncycloplegic autorefraction. Students were defined as eligible for correction if presenting with functional visual impairment (uncorrected VA >0.2 logMAR) accompanied by myopia (≤−0.50 diopters [D]), hyperopia (≥+2.00 D), or astigmatism (≥0.75 D). Refractive error coverage was calculated as the proportion of eligible students possessing vision-improving glasses. A nested questionnaire substudy (*n* = 17,014) evaluated multidimensional sociodemographic determinants. Predictors of coverage were identified using both multivariate logistic regression and a random forest machine learning algorithm.

**Results:**

Of the 1,113,548 students, 41.26% were eligible for correction, and the overall refractive error coverage was 57.92%. Coverage was significantly positively associated with educational level and inversely with uncorrected visual acuity (UCVA) severity (*P* < 0.001). Logistic regression revealed higher coverage was significantly associated with female gender, non-left-behind status (odds ratio = 2.30), better academic performance, higher parental education, and parental glasses wear. Random forest analysis identified bilateral UCVA and grade level as the top predictors of coverage.

**Conclusions:**

Refractive error coverage among Hubei students remains suboptimal. Improving these rates requires targeted public health interventions prioritizing males, lower-grade students, and rural/left-behind children, alongside initiatives to enhance parental eye health literacy.

**Translational Relevance:**

By translating data from over 1.1 million students into predictive models, this study provides a robust, evidence-based foundation to optimize targeted clinical vision screening protocols for vulnerable pediatric populations.

## Introduction

In 2020, uncorrected refractive error was the leading cause of moderate and severe visual impairment worldwide.^1^^,^^2^ School-aged children and adolescents are at crucial stages for the occurrence and development of refractive error, particularly with myopia. In these young populations, uncorrected refractive error significantly contributes to avoidable visual impairment,^3^ yet it can be effectively corrected using simple methods such as glasses and contact lenses.^4^^–^^6^ Although studies by the Vision Loss Expert Group^7^ have shown that the estimated distance effective refractive error coverage (eREC) and near vision eREC in individuals aged 50 and above are 42.9% and 20.5%, respectively, research focusing on the refractive error coverage in school-aged children and adolescents is relatively scarce.

Both myopia and hyperopia, impairing the ability to see distant and nearby objects, respectively, have been proven to impact academic performance negatively.^8^^,^^9^ In the high-pressure educational climate of China, such visual impairments may exacerbate anxiety and depression, particularly as children are affected for longer durations.^10^^,^^11^ Vision impairment diminishes quality of life by limiting daily activities and increasing the risk of accidents, including in traffic,^12^^,^^13^ and adversely affects children's cognitive development, psychological state, and mental health. Timely correction of refractive error is crucial as it can significantly improve cognitive and educational outcomes, enhance psychological well-being and overall quality of life, and prevent the progression to irreversible vision impairment.^14^^–^^17^

To address this gap, we leveraged an unprecedented, population-based dataset encompassing over 1.1 million students in Hubei Province, China, to conduct a comprehensive assessment of refractive error coverage. Moving beyond conventional descriptive epidemiology, this study innovatively integrates traditional multivariate analysis with a random forest machine learning algorithm to quantify the relative importance of multidimensional sociodemographic and clinical factors. Ultimately, this study aims to translate large-scale, real-world data into actionable insights, providing robust evidence for precision public health interventions and targeted resource allocation in pediatric eye care.

## Methods

### Study Population

This study is based on a population-based prospective cross-sectional survey initiated in 2022, targeting students from kindergartens, primary schools, junior high schools, regular high schools, and vocational high schools across 103 county-level administrative regions in Hubei Province. Annually, from October to November, a stratified cluster sampling is conducted based on schools, grades, and classes, with a minimum sampling requirement of one regular high school, two junior high schools, two primary schools, and two kindergartens in each county-level administrative region, with a minimum of 80 students per grade. For municipal districts, an additional regular high school and vocational high school are included. To date, three annual surveys covering the entire province have been completed. In total, 1,152,284 data entries from the three surveys have been preliminarily included in this study. Among them, 20,316 students with poor vision caused by factors such as pediatric eye diseases, corneal diseases, and retinal diseases unrelated to refractive error were excluded. Additionally, 11,652 students lacking vision or refractive data were excluded. The detailed inclusion and exclusion criteria are summarized in Table 1.

Furthermore, since some schools were randomly sampled multiple times over the 3 years, we used the students’ unique ID numbers to identify and remove duplicate records, retaining only the most recent screening data for students with multiple entries to ensure sample independence. The survey now encompasses data from 486 kindergartens with 62,080 students, 687 primary schools with 556,313 students, 393 junior high schools with 258,862 students, 291 regular high schools with 195,399 students, and 76 vocational high schools with 40,894 students. In total, the study includes 1,113,548 students across 1933 schools. Screenings were conducted classwide during regular school hours. Although individual absentee logs were not tracked due to the cohort's massive scale, the mandatory nature of schooling yielded an effective participation rate exceeding 95% (aligning with daily attendance), thereby minimizing selection bias. Furthermore, to streamline data collection across this extensive cluster sample, demographic data were aggregated by grade level rather than individual age. The corresponding estimated age ranges are as follows: kindergarten (3–6 years); primary school, grades 1–6 (6–12 years); junior high school, grades 7–9 (12–15 years); and senior/vocational high school (15–18 years).

### Ethics

This study adheres to the principles outlined in the Declaration of Helsinki and has obtained ethical approval from the Ethics Committee of Renmin Hospital of Wuhan University (WDRY2020-K211). Because the participants are minors, prior to the commencement of any screening or survey activities, written informed consent was obtained from their parents or legal guardians after they were provided with detailed information about the study. Additionally, verbal assent was obtained from the students themselves on the day of the examination.

### Information Management System

We established an information management system during the preparation phase. This system is a dedicated database built on Microsoft SQL Server 2008 (Microsoft, Redmond, WA, USA), designed for uploading, storing, and managing screening data. Before the commencement of screening, personal information of each student, including full name, gender, ID number, school name, grade, class, and administrative division, was entered into the system. To facilitate on-site data management, each student was assigned a system-generated QR code for real-time record access and verification during the screening. While a new QR code may be issued for subsequent annual events, it remains permanently anchored to the student's unique identification number within the backend database. This enables accurate longitudinal tracking and allows for rigorous deduplication of records across multiple survey years. Subsequently, data collected from autorefractors and visual acuity tests are uploaded in real time to the system via a wireless network or Bluetooth devices.

### Ophthalmic Examination

#### Distance Visual Acuity Examination

Distance visual acuity (DVA) examinations were performed using the 5-m standard logarithmic visual sharpness E-chart. To ensure standardized measurements across all schools, examinations were conducted in appropriately illuminated indoor settings, with the testing distance strictly marked at 5 m. Uniformly trained ophthalmologists or optometrists followed standardized operational instructions. Furthermore, to ensure measurement accuracy among younger participants (e.g., kindergarteners), school teachers were instructed to conduct E-chart familiarization sessions on the day prior to the examination, ensuring that these children could correctly identify and indicate the optotype orientations. On the day of screening, participants who used vision correction were instructed to wear their usual glasses. Both uncorrected distance visual acuity (UDVA) and presenting DVA were measured, and the type of glasses worn (glasses, contact lenses, orthokeratology lenses) was recorded. For those not wearing glasses, only UDVA was measured. Before the examination, screening personnel scanned the QR code of each screening participant using a mobile phone to access the visual acuity examination interface. The examinee was positioned 5 m from the visual acuity chart, and the visual acuity was recorded using the 5-point logarithmic visual acuity system, which is the national standard in China. For international accessibility, the 5-point score can be directly converted to the logarithm of the minimum angle of resolution (logMAR) scale using the following formula: logMAR = 5.0 – (5-point score). For example, a standard normal vision of 5.0 corresponds to 0.0 logMAR (20/20 Snellen). The results of the visual acuity examination are automatically uploaded in real time to the information management system through wireless networks or Bluetooth.

#### Autorefraction

Before the examination, screening personnel scanned the QR code of each participant using a mobile phone to access the interface for autorefractor examination. The autorefractor examination was conducted using the same model of autorefractor globally (TOPCON KR-800; Topcon, Tokyo, Japan) to eliminate interinstrument variability. Daily calibration was performed using a standard model eye before screening commenced. For each participant, three measurements were taken for each eye. If the spherical or cylindrical power difference between any two measurements was ≥0.50 diopters (D), additional measurements were taken until the readings stabilized. Upon completion, the average results from the autorefractor examination were instantly uploaded to the information management system using wireless networks or Bluetooth. The details of the standardized equipment utilized in the screening program are presented in Table 2.

### Definition and Calculation Method for Refractive Error Coverage

**A** = the number of students with uncorrected visual acuity of less than 4.8 (equivalent to >0.2 logMAR or worse than 20/32 Snellen) in either eye along with myopia of −0.5 D or less, hyperopia of +2.0 D or greater, or astigmatism of 0.75 D or greater

**B** = the number of students (among those in group A) who wear glasses at school and achieve better visual acuity through wearing glasses^18^^,^^19^

$$\begin{array}{c} \mathrm{Refractive}\;\mathrm{error}\;\mathrm{coverage}=\left(B/A\right)\times 100\% \end{array}$$

### Individual and Sociodemographic Factors Survey

Rather than surveying the entire 1.1 million cohort, the questionnaire survey was designed as a nested, targeted substudy using stratified cluster random sampling to evaluate sociodemographic factors. During the screening, questionnaires were distributed using a stratified random sampling approach. Specifically, two primary schools, one junior high school, and one regular high school were randomly selected from each of Hubei's 17 administrative divisions (13 prefectures and 4 municipalities). In this study, the final analysis was based on a total of 17,014 valid and fully completed questionnaires returned by students in grades 4 to 6, junior high, and regular high schools. The family, school, and individual factors involved in this study specifically include urban or rural residence, grade level, gender, parental glasses wear, parental education, parental knowledge of eye health, only-child status, left-behind child status, presence of myopic friends, and academic performance. Urban or rural classification follows the administrative divisions of Hubei Province. Grade and gender were obtained upon inputting student information into the information management system, while the remaining factors were collected through questionnaire surveys (see Supplementary Material Appendix A questionnaire survey English translation). Specifically, “left-behind” status was defined as children under 18 years of age who remained in their rural hometowns while one or both parents migrated to other areas for work for at least 6 months.^20^ The questionnaires were paper-based and distributed by school teachers. To minimize self-reporting bias and ensure data reliability, students were required to take the questionnaires home and complete them with the assistance of their parents or legal guardians before returning them to the school.

### Statistical Analysis

We used R software version 4.2.2 for statistical data analysis. To handle missing data, a complete case analysis approach was adopted; any records with missing values for the variables of interest were excluded from the final analytical dataset. The total number of participants screened, the number of participants with refractive errors eligible for correction, and refractive error coverage were described using counts (percentages). To explore the factors influencing refractive error coverage, we conducted single-factor analysis using the χ^2^ test. Further, a multiple-factor logistic regression was performed, treating refractive error coverage as the dependent variable. A significance level of *P* < 0.05 was used to determine statistical significance. Additionally, a random forest machine learning algorithm was applied to investigate the importance of related factors. Additionally, a random forest machine learning algorithm was applied to investigate the importance of related factors. While traditional statistical analyses were performed using R software, the random forest model was implemented using the scikit-learn library in Python. To rigorously prevent overfitting, the model's hyperparameters were strictly constrained. Finally, to identify the top predictors of refractive error coverage, variable importance was assessed using “mean decrease accuracy.” This metric measures the reduction in the model's overall predictive accuracy when the data for a specific variable are randomly permuted (shuffled).

## Results

**Figure 1.**
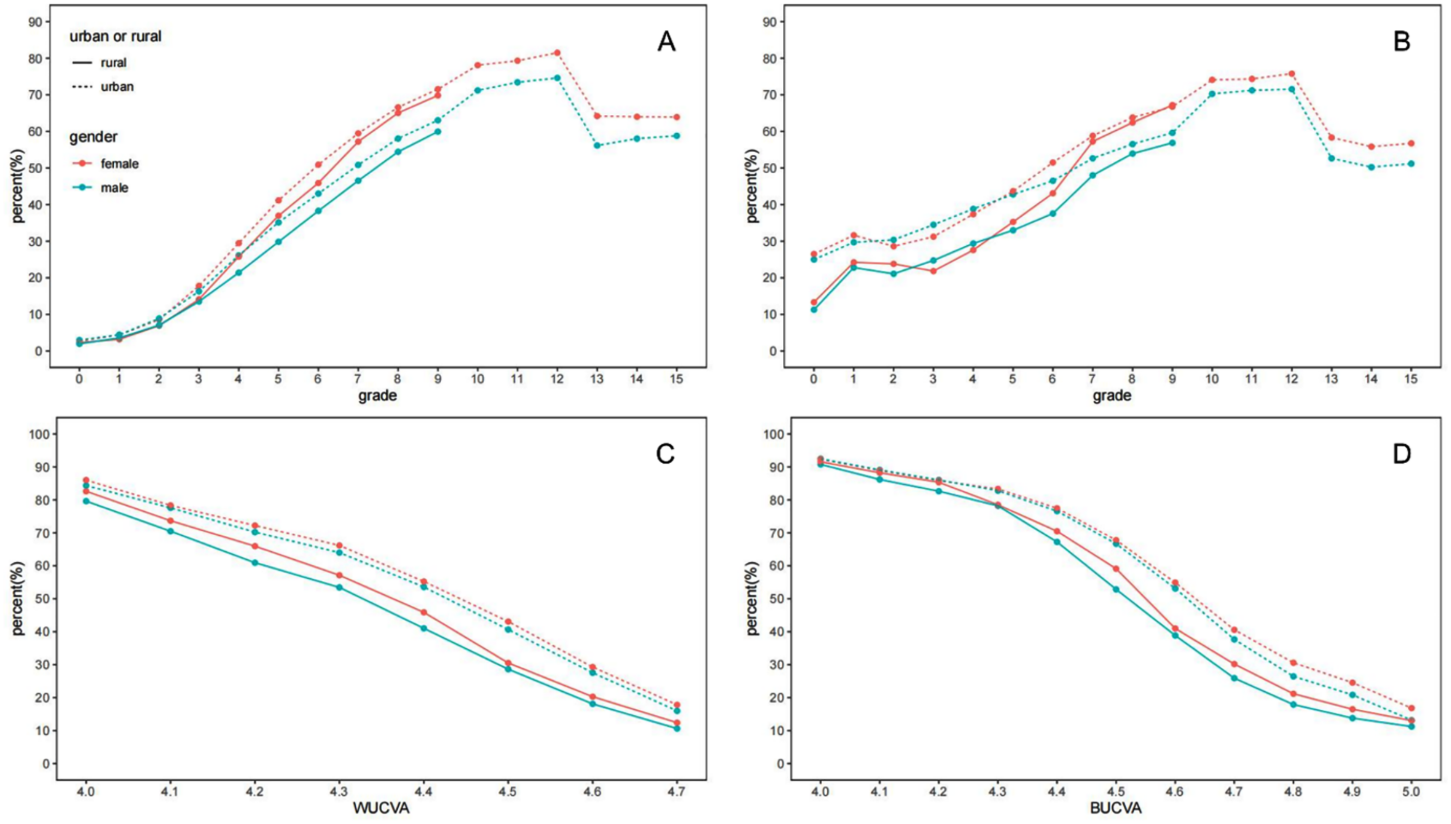
Line graphs showing (**A**) the eligibility for correction of refractive errors varies with grade, (**B**) the refractive error coverage varies with grade, (**C**) the refractive error coverage varies with WUCVA, and (**D**) the refractive error coverage varies with BUCVA. 0: kindergarten; 1–6: primary school; 7–9: junior high school; 10–12: regular high school; 13–15: vocational high school.

This study encompassed a total of 1,113,548 screening participants, comprising 588,682 males and 514,866 females. The number of individuals with refractive error eligible for correction was 459,454 (41.26%), with a higher rate among females (43.97%) compared to males (38.93%) (^2^Table 3). The proportion of students eligible for refractive correction increased progressively with educational level, rising from 2.67% among kindergarteners to 76.04% among regular high school students. Interestingly, the proportion of students eligible for refractive correction among vocational high school students was lower than that among junior high school students. The rate of refractive error eligible for correction in rural areas (29.42%) was significantly lower than in urban areas (40.33%) (Fig. 1A).

**Table 1.** Inclusion and Exclusion Criteria

Inclusion Criteria	Exclusion Criteria
• Official enrollment and attendance at selected schools during the screening period • Clear consciousness, normal cognitive function, and ability to cooperate with ophthalmic examinations • Voluntary participation with written informed consent from parents or legal guardians	• Ocular diseases affecting visual acuity (e.g., corneal or fundus diseases, strabismus, amblyopia) • History of ocular surgery • Severe systemic diseases or physical disabilities • Psychiatric disorders, history of antipsychotic medication use, or recent major psychological trauma • Poor compliance, inability to cooperate with eye examinations, or inability to complete the questionnaire • Lack of visual acuity or refractive data

**Table 2.** Standardized Equipment Utilized in the Screening Program

Examination	Equipment	Manufacturer/Location	Key Protocol
Distance visual acuity	5-m Standard Logarithmic E chart	Standardized domestic medical device	5-m distance, standardized indoor illumination
Autorefraction	TOPCON KR-800 Autorefractor	Topcon Corporation, Tokyo, Japan	Same model used at all sites; daily calibration with model eye; variance ≤0.50 D required

**Table 3.** Status of School Students Eligible for Correction for Refractive Error

Characteristic	Overall (*n* = 1,113,548), *n* (%)	Eligible for Correction (Group A) (*n* = 459,454), *n* (%)	Not Eligible (*n* = 654,094), *n* (%)	*P* Value^a^
Educational level				<0.001
Kindergarten	62,080	1656 (2.67)	60,424 (97.33)	
Primary school	556,313	128,014 (23.01)	428,299 (76.99)	
Junior high school	258,862	156,521 (60.47)	102,341 (39.53)	
Regular high school	195,399	148,582 (76.04)	46,817 (23.96)	
Vocational high school	40,894	24,681 (60.35)	16,213 (39.65)	
Urban or rural				<0.001
Urban	938,278	407,890 (43.47)	530,388 (56.53)	
Rural	175,270	51,564 (29.42)	123,706 (70.58)	
Gender				<0.001
Male	598,682	233,093 (38.93)	365,589 (61.07)	
Female	514,866	226,361 (43.97)	288,505 (56.03)	

a Pearson's χ^2^ test.

This study included a total of 459,454 screening participants eligible for refractive error correction. Among them, 266 134 (57.92%) received refractive error correction, with a higher refractive error coverage among females (60.22%) compared to males (55.69%) (Table 4). As the educational level increased, the refractive error coverage gradually increased from 23.49% during kindergarten to 72.80% during regular high school. However, the refractive error coverage among vocational high school students (54.00%) was lower than that among junior high school students (59.40%) (Fig. 1B). Coverage rates demonstrated a strong inverse relationship with uncorrected visual acuity (UCVA). For instance, coverage among students with a worse-seeing eye UCVA of 4.5 to 4.7 was 27.83%, increasing to 61.51% for a UCVA of 4.2 to 4.4 and peaking at 82.92% for a UCVA of 4.1 or lower (Fig. 1C). Conversely, coverage was only 20.38% for those with a better-seeing eye UCVA of 4.8 to 5.0 (Fig. 1D). Additionally, the refractive error coverage increased with the severity of myopia—from 35.42% for low myopia to 90.56% for high myopia. The refractive error coverage among hyperopes (41.80%) was higher than that among students with low myopia. The refractive error coverage among individuals with astigmatism (72.66%) was higher than that among those without astigmatism (53.73%). Urban students showed a higher refractive error coverage (59.38%) compared to their rural counterparts (46.45%).

**Table 4.** Refractive Error Coverage and its Influencing Factors

Characteristic	Overall (*n* = 459,454), *n* (%)	Corrected (*n* = 266,134), *n* (%)	Uncorrected (*n* = 193,320), *n* (%)	*P* Value^a^
Educational level				<0.001
Kindergarten	1656	389 (23.49)	1267 (76.51)	
Primary school	128,014	51,275 (40.05)	76,739 (59.95)	
Junior high school	156,521	92,975 (59.40)	63,546 (40.60)	
Regular high school	148,582	108,168 (72.80)	40,414 (27.20)	
Vocational high school	24,681	13,327 (54.00)	11,354 (46.00)	
Gender				<0.001
Male	233,093	129,820 (55.69)	103,273 (44.31)	
Female	226,361	136,314 (60.22)	90,047 (39.78)	
WUCVA				<0.001
3.3 to 4.1 (logMAR 1.7 to 0.9)	162,385	134,657 (82.92)	27,728 (17.08)	
4.2 to 4.4 (logMAR 0.8 to 0.6)	144,889	89,126 (61.51)	55,763 (38.49)	
4.5 to 4.7 (logMAR 0.5 to 0.3)	152,180	42,351 (27.83)	109,829 (72.17)	
BUCVA				<0.001
3.3 to 4.1 (logMAR 1.7 to 0.9)	86,815	79,203 (91.23)	7612 (8.77)	
4.2 to 4.4 (logMAR 0.8 to 0.6)	111,874	90,282 (80.70)	21,592 (19.30)	
4.5 to 4.7 (logMAR 0.5 to 0.3)	139,491	71,935 (51.57)	67,556 (48.43)	
4.8 to 5.0 (logMAR 0.2 to 0.0)	121,274	24,714 (20.38)	96,560 (79.62)	
Refractive status				<0.001
Mild myopia	209,891	74,338 (35.42)	135,553 (64.58)	
Moderate myopia	198,361	148,867 (75.05)	49,494 (24.95)	
High myopia	44,151	39,982 (90.56)	4169 (9.44)	
Hyperopia	7051	2947 (41.80)	4104 (58.20)	
Astigmatism				<0.001
Nonastigmatism	357,705	192,205 (53.73)	165,500 (46.27)	
Astigmatism	101,749	73,929 (72.66)	27,820 (27.34)	
Urban or rural				<0.001
Urban	407,890	242,185 (59.38)	165,705 (40.62)	
Rural	51,564	23,949 (46.45)	27,615 (53.55)	

a Pearson's χ^2^ test.

Among the 17,014 questionnaire surveys included in this study, 8540 individuals were eligible for correction for refractive error, of whom 5279 (61.81%) received correction.

In the univariate analysis, 10 variables met the criteria of *P* ≤ 0.2 and were subsequently included in the multivariate logistic regression model: worse-seeing eye's uncorrected visual acuity (WUCVA), better-seeing eye's uncorrected visual acuity (BUCVA), educational level, gender, urban–rural residency, parental glasses wear, parental eye health knowledge, left-behind child status, parents with >12 years of education, and academic performance. Variables including being an only child (*P* = 0.3) and having myopic friends (*P* = 0.3) were excluded from the multivariate model.

In the multivariate analysis, higher refractive error coverage was significantly associated with female gender (odds ratio [OR] = 1.16; 95% confidence interval [CI], 1.03–1.30; *P* = 0.012), children who were not left behind (OR = 2.30; 95% CI, 1.87–2.82; *P* < 0.001), having parents who wear glasses (OR = 1.32; 95% CI, 1.17–1.49; *P* < 0.001), parents with >12 years of education (OR = 1.84; 95% CI, 1.56–2.18; *P* < 0.001), and students in the top 50% academically (OR = 1.14; 95% CI, 1.02–1.28; *P* = 0.023). Conversely, parental eye health knowledge and urban–rural residency lost their significance in the multivariate model (both *P* > 0.05) (Table 5).

**Table 5.** Logistic Regression Analysis of Factors Influencing Refractive Error Coverage

				Multivariate Model^b^		
Characteristic	Corrected (*n* = 5279), *n* (%)	Uncorrected (*n* = 3261), *n* (%)	*P* Value^a^	OR	95% CI	*P* Value
≥1 parent wears glasses			<0.001			
No	2779 (57.68)	2039 (42.32)		—	—	
Yes	2500 (67.17)	1222 (32.83)		1.32	1.17–1.49	<0.001
Eye health knowledge			<0.001			
No	1144 (58.16)	823 (41.84)		—	—	
Yes	4135 (62.91)	2438 (37.09)		0.97	0.85–1.11	0.7
Left-behind child			<0.001			
Yes	298 (41.74)	416 (58.26)		—	—	
No	4981 (63.65)	2845 (36.35)		2.30	1.87–2.82	<0.001
Only child in family			0.3			
No	3384 (61.40)	2127 (38.60)				
Yes	1895 (62.56)	1134 (37.44)				
≥1 parent with >12 y of education			0.2			
No	4367 (61.49)	2735 (38.51)		—	—	
Yes	912 (63.42)	526 (36.58)		1.84	1.56–2.18	<0.001
Myopic friends			0.3			
No	2077 (61.18)	1318 (38.82)				
Yes	3202 (62.24)	1943 (37.76)				
Top 50% academic performance			<0.001			
No	2283 (58.27)	1635 (41.73)		—	—	
Yes	2996 (64.82)	1626 (35.18)		1.14	1.02–1.28	0.023
Educational level			<0.001			
Primary school	848 (39.92)	1276 (60.08)		—	—	
Junior high school	1302 (60.17)	862 (39.83)		1.51	1.29–1.77	<0.001
Regular high school	3129 (73.59)	1123 (26.41)		1.86	1.60–2.17	<0.001
Gender			0.025			
Male	2307 (60.50)	1506 (39.50)		—	—	
Female	2972 (62.87)	1755 (37.13)		1.16	1.03–1.30	0.012
Urban and rural			<0.001			
Rural	519 (49.15)	537 (50.85)		—	—	
Urban	4682 (64.53)	2573 (35.47)		1.17	0.98–1.40	0.077
WUCVA			<0.001			
4.5–4.7	632 (25.95)	1803 (74.05)		—	—	
4.2–4.4	1447 (61.37)	911 (38.63)		2.07	1.78–2.41	<0.001
3.3–4.1	3200 (85.40)	547 (14.60)		3.21	2.66–3.88	<0.001
BUCVA			<0.001			
4.8–5.0	379 (18.87)	1630 (81.13)		—	—	
4.5–4.7	1160 (51.62)	1087 (48.38)		4.73	4.07–5.52	<0.001
4.2–4.4	1694 (81.56)	383 (18.44)		11.2	9.34–13.5	<0.001
3.3–4.1	2046 (92.71)	161 (7.29)		24.2	19.0–30.9	<0.001

a Pearson's χ^2^ test.

b Multivariate model including all the variables significant at *P* < 0.2 in univariate analysis.

**Figure 2.**
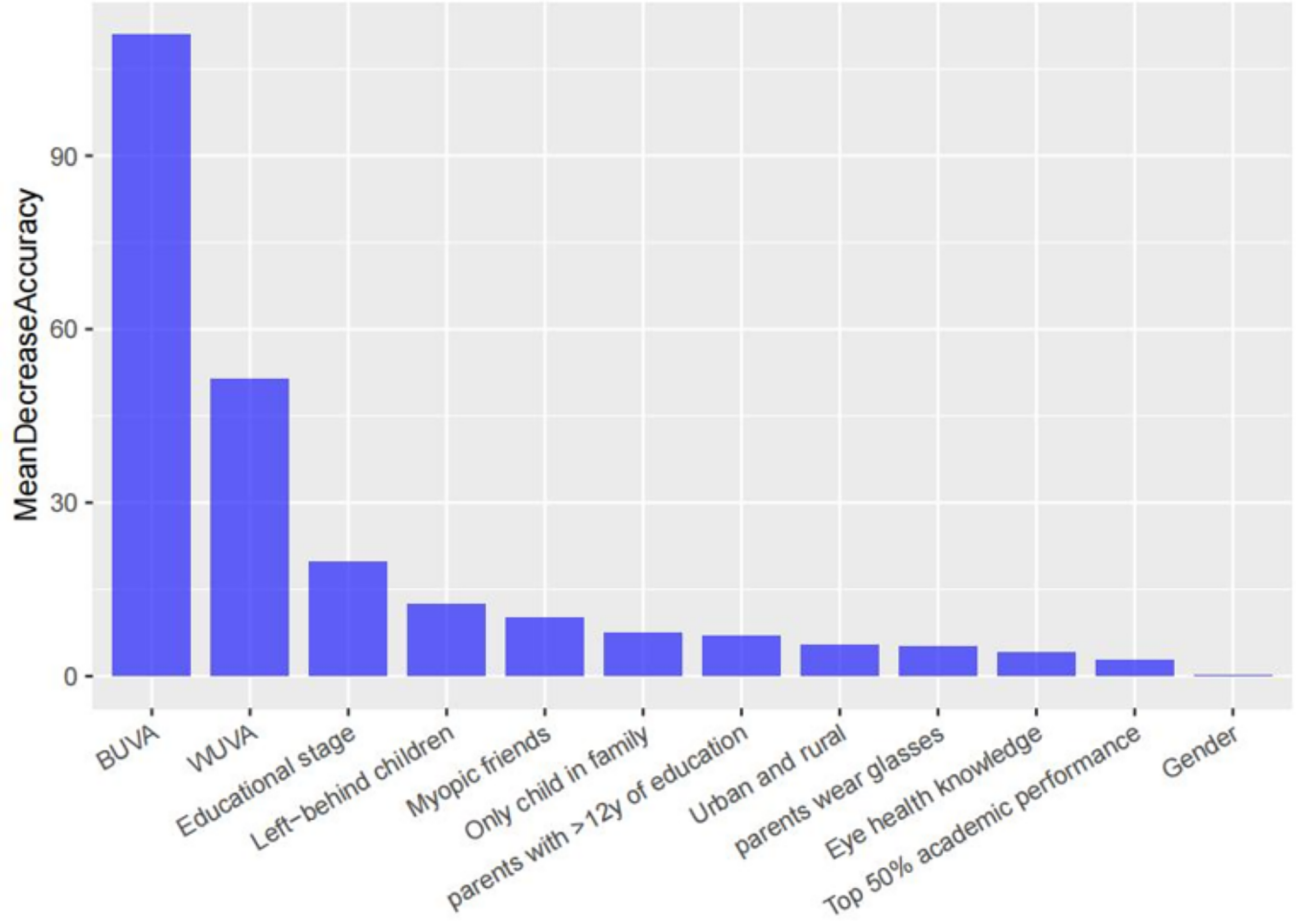
Variable importance in random forest.

In our study, we used the random forest algorithm to evaluate the importance of factors on the refractive error coverage. The random forest model had an area under the curve of 0.85. BUCVA, WUCVA, and educational stage were the top three factors influencing the refractive error coverage (Fig. 2).

## Discussion

Leveraging a massive real-world dataset of over 1.1 million school-aged children, this study provides a highly representative evaluation of refractive error coverage. Our findings reveal an overall coverage rate of 57.92% among students requiring correction in Hubei Province. By utilizing both logistic regression and random forest machine learning models, we robustly identified that the severity of uncorrected visual acuity and educational stage are the paramount drivers of coverage. Furthermore, our multidimensional analysis highlights significant sociodemographic disparities: higher coverage is significantly associated with female gender, urban residency, higher parental education, parental glasses wear, non-left-behind status, and superior academic performance. These findings underscore the urgent need to translate epidemiologic evidence into targeted screening and intervention policies, particularly prioritizing vulnerable subgroups such as rural, left-behind, and male students with early-stage vision impairment.

Due to differences in the study areas, ages, and definitions of refractive error coverage, there is significant variation in refractive error coverage among different studies.^21^^–^^25^ For example, a study on school-age children in Shantou, China, found that 60.0% of myopic students, defined as having spherical equivalent refraction <−0.50 D in any eye and UCVA <20/20, did not receive refractive correction.^26^ In studies focusing on rural and urban migrant students in China, refractive error coverage was determined based on a threshold of uncorrected visual acuity ≤6/12 in either eye, resulting in lower coverage rates of 37.0% for females and 34.7% for males.^18^^,^^27^ This is significantly lower than the 46.45% coverage observed in rural areas in our study. Additionally, a comparative study among junior high school students in the Ningxia Hui Autonomous Region reported a refractive error coverage of 56.8% among Han ethnic students, slightly lower than the 59.40% observed in our study. In contrast, the coverage for Hui ethnic students was only 41.5%.^28^ These results highlight significant differences in refractive error coverage among different regions and populations, which may be related to factors such as regional economy and ethnic distribution.

Previous studies confirmed the economic level as an essential factor influencing refractive error coverage.^29^ Whether in high-income areas or among high-income populations, refractive error coverage is relatively higher compared to low-income areas or populations, and the incidence of visual impairment and blindness is also lower in high-income areas.^30^ This correlation is supported by our findings, which show higher refractive error coverage among students in urban schools than in rural areas.^18^ The economic advantages of urban areas likely contribute to this difference. Urban regions benefit from greater government health care expenditures and offer residents superior medical services and more convenient access to ophthalmic examinations and treatments.^29^ On the other hand, residents in urban areas pay more attention to their health and have a better understanding of relevant eye health knowledge. Therefore, efforts should be made to improve the accessibility of eye health services in low-income areas to increase the refractive error coverage.

Our study found that visual acuity and refractive status are key factors influencing refractive error coverage,^31^ which is consistent with previous research findings. Uncorrected refractive errors lead to blurred vision and are associated with decreased vision-related quality of life. Declining vision can impact students’ ability to see the blackboard, causing attention distraction, reduced classroom participation, and ultimately a decline in academic performance, with more severe vision impairments intensifying these effects.^32^^–^^34^ As educational grades increase, students face heavier academic burdens and higher demands on visual quality, thus increasing the refractive error coverage. Additionally, declining vision also increases the risk of injury during physical activities. To mitigate this risk, individuals with impaired vision may reduce physical activity and increase sedentary time.^35^ Similarly, the impact of astigmatism on vision-related quality of life is more significant, with students having this condition tending to correct refractive error.^36^

Our study results show that students ranking in the top 50% academically exhibit higher refractive error coverage. We also found that the refractive error coverage in vocational high schools is significantly lower than in regular high schools and even lower than in junior high schools, which further corroborates the influence of academic performance on refractive error coverage. In China, students with lower academic achievements often enroll in vocational high schools, while those with better academic performance typically attend regular high schools. Students with better academic performance spend more time engaged in near-work and require clearer distant vision, and refractive correction also has a positive impact on academic performance. Contrary to some previous research, our study suggests that having myopic friends does not significantly influence refractive error coverage.^23^ Teachers’ attitudes and knowledge about eye care also play an essential role in refractive error coverage.^37^^,^^38^ If teachers have relevant knowledge about refractive error and support correction, the refractive error coverage among students will significantly increase. Encouragement and support from teachers, especially in purchasing and wearing glasses, positively impact students’ correction decisions.

Research results indicate that parents play a pivotal role in refractive error coverage among children and adolescents. When parents wear glasses, they pay more attention to their children's eye health and possess more eye health knowledge, leading to a higher refractive error coverage among children. Conversely, when parents work outside the home, and grandparents take care of children, the refractive error coverage among children is lower due to grandparents’ insufficient knowledge of eye health. This finding emphasizes the critical role of parents in cultivating good eye health behaviors in children.^18^^,^^27^

In response to the current low refractive error coverage, several effective measures can be implemented.^39^ Research has shown that providing free eyeglasses during vision screenings significantly enhances refractive error coverage, as opposed to merely providing prescriptions for glasses.^40^^,^^41^ For example, a study in India found that distributing ready-made eyeglasses decreased the visual impairment rate among students and reduced the costs associated with school-based eye health programs.^41^ Additionally, a study in China demonstrated that establishing vision centers in county hospitals can effectively increase refractive error coverage.^42^ Increasing societal awareness of refractive error correction and encouraging parents and students to actively participate in eye health management are of great significance in improving refractive error coverage.

Compliance with wearing glasses is a crucial factor in addressing preventable visual impairment and blindness.^43^^,^^44^ A study conducted in India found that 20% of school students do not wear their prescribed glasses as recommended.^45^ Research on preschool children indicates that approximately 75% demonstrate good compliance with wearing glasses.^46^^,^^47^ The willingness to wear glasses regularly is influenced by factors such as binocular uncorrected visual acuity, monocular refractive error, and the perceived impact of glasses on personal appearance.^48^ Regarding methods to improve compliance with wearing glasses, incentivizing students through teacher rewards and having teachers supervise students’ wearing of glasses is a feasible approach.^49^ Additionally, integrating technology such as the SpecsOn monitoring devices (The University of Auckland, Auckland, New Zealand) can improve compliance with wearing glasses.^50^ These devices can detect whether glasses are being worn properly and consistently, providing a direct method for monitoring and encouraging compliance.

This study is a cross-sectional study based on the school population, covering 103 county-level administrative regions in Hubei Province over 3 years. The sample size is adequate and representative. Both vision and optometry data were collected by professional ophthalmologists following standard procedures, ensuring the accuracy of the results. We combined publicly available data with individual questionnaire surveys and utilized machine learning to analyze the socioeconomic factors and personal factors influencing refractive error coverage.

Several limitations of this study should be acknowledged. First, the use of noncycloplegic refraction—combined with UCVA to determine refractive status—is a primary limitation. Noncycloplegic autorefraction is known to overestimate the prevalence and severity of myopia while underestimating hyperopia, as active accommodation can induce a more negative refractive power. Previous research has demonstrated a significant shift in the mean spherical equivalent when comparing noncycloplegic and cycloplegic results.^51^ In the context of our study, this accommodation-induced bias may have artificially inflated the number of students identified as eligible for correction (group A). Consequently, the overall refractive error coverage (B/A) reported here may represent an underestimation of the actual coverage, as many “pseudo-myopic” students included in the denominator would not clinically require or seek glasses correction. Future studies utilizing cycloplegic refraction in a representative subsample would be highly beneficial to precisely quantify the exact magnitude of this overestimation within the Hubei student population. Additionally, due to the massive scale of the screening cohort, assessing the best-corrected visual acuity for all study participants was logistically unfeasible. Second, our operational definition for hyperopic correction relied on a fixed threshold of ≥+2.0 D combined with a UCVA <4.8. While aligning with standardized epidemiologic protocols ensures comparability and effectively uses the UCVA filter to exclude young, asymptomatic hyperopes with sufficient accommodative amplitude, this uniform threshold has inherent clinical drawbacks. It cannot account for personalized clinical variables, such as binocular vision anomalies or asthenopia, particularly in older students. Consequently, our study may underestimate the specific refractive needs of symptomatic students with lower degrees of hyperopia. Finally, there are limitations related to demographic sampling and questionnaire administration. Because regular high schools are predominantly located in urban areas, we did not collect samples from high school students residing in rural areas. Furthermore, the questionnaire survey was conducted as a targeted substudy utilizing take-home paper forms, an approach inherently susceptible to logistical challenges and response bias.

Refractive correction coverage among students in Hubei Province is 57.92%. Characteristics of individuals lacking refractive correction include being male, residing in rural areas, being in a lower grade level, being left-behind children, and experiencing mild visual impairment. Future vision control efforts should focus on these groups to increase refractive errors coverage.

## Supplementary Material

Supplement 1

## References

[1] GBD 2019 Blindness and Vision Impairment Collaborators; Vision Loss Expert Group of the Global Burden of Disease Study. Trends in prevalence of blindness and distance and near vision impairment over 30 years: an analysis for the Global Burden of Disease Study. *Lancet Glob Health*. 2021; 9(2): e130–e143. 33275950 10.1016/S2214-109X(20)30425-3 PMC7820390

[2] GBD 2019 Blindness and Vision Impairment Collaborators; Vision Loss Expert Group of the Global Burden of Disease Study. Causes of blindness and vision impairment in 2020 and trends over 30 years, and prevalence of avoidable blindness in relation to VISION 2020: the Right to Sight: an analysis for the Global Burden of Disease Study. *Lancet Glob Health*. 2021; 9(2): e144–e160. 33275949 10.1016/S2214-109X(20)30489-7 PMC7820391

[3] Xu T, Wang B, Liu H, et al. Prevalence and causes of vision loss in China from 1990 to 2019: findings from the Global Burden of Disease Study 2019. *Lancet Public Health*. 2020; 5(12): e682–e691. 33271081 10.1016/S2468-2667(20)30254-1

[4] Chen M, Xu L, Li H, et al. Myopia control with multifocal lens in school-aged children: a meta-analysis. *Front Pediatr*. 2022; 10: 889243. 35795335 10.3389/fped.2022.889243 PMC9251339

[5] Bao J, Huang Y, Li X, et al. Spectacle lenses with aspherical lenslets for myopia control vs single-vision spectacle lenses: a randomized clinical trial. *JAMA Ophthalmol*. 2022; 140(5): 472–478. 35357402 10.1001/jamaophthalmol.2022.0401 PMC8972151

[6] Wildsoet CF, Chia A, Cho P, et al. IMI—Interventions Myopia Institute: interventions for controlling myopia onset and progression report. *Invest Ophth Vis Sci*. 2019; 60(3): M106–M131. 10.1167/iovs.18-25958 30817829

[7] Bourne R, Cicinelli MV, Sedighi T, et al. Effective refractive error coverage in adults aged 50 years and older: estimates from population-based surveys in 61 countries. *Lancet Glob Health*. 2022; 10(12): e1754–e1763. 36240807 10.1016/S2214-109X(22)00433-8

[8] Ma X, Zhou Z, Yi H, et al. Effect of providing free glasses on children's educational outcomes in China: cluster randomized controlled trial. *BMJ*. 2014; 349: g5740. 25249453 10.1136/bmj.g5740 PMC4172821

[9] Neitzel AJ, Wolf B, Guo X, et al. Effect of a randomized interventional school-based vision program on academic performance of students in grades 3 to 7: a cluster randomized clinical trial. *JAMA Ophthalmol*. 2021; 139(10): 1104–1114. 34499111 10.1001/jamaophthalmol.2021.3544 PMC8430909

[10] Li D, Chan VF, Virgili G, et al. Impact of vision impairment and ocular morbidity and their treatment on depression and anxiety in children: a systematic review. *Ophthalmology*. 2022; 129(10): 1152–1170. 35660416 10.1016/j.ophtha.2022.05.020

[11] Bazarczyk JB, Urban B, Konarzewska B, et al. The differences in level of trait anxiety among girls and boys aged 13-17 years with myopia and emmetropia. *BMC Ophthalmol*. 2016; 16(1): 201. 27842529 10.1186/s12886-016-0382-2 PMC5109705

[12] Piyasena P, Olvera-Herrera VO, Chan VF, et al. Vision impairment and traffic safety outcomes in low-income and middle-income countries: a systematic review and meta-analysis. *Lancet Glob Health*. 2021; 9(10): e1411–e1422. 34411516 10.1016/S2214-109X(21)00303-X

[13] Rodriguez-Ayllon M, Cadenas-Sánchez C, Estévez-López F, et al. Role of physical activity and sedentary behavior in the mental health of preschoolers, children and adolescents: a systematic review and meta-analysis. *Sports Med*. 2019; 49(9): 1383–1410. 30993594 10.1007/s40279-019-01099-5

[14] Assi L, Chamseddine F, Ibrahim P, et al. A global assessment of eye health and quality of life: a systematic review of systematic reviews. *JAMA Ophthalmol*. 2021; 139(5): 526–541. 33576772 10.1001/jamaophthalmol.2021.0146 PMC7881366

[15] Pirindhavellie GP, Yong AC, Mashige KP, Naidoo KS, Chan VF. The impact of spectacle correction on the well-being of children with vision impairment due to uncorrected refractive error: a systematic review. *BMC Public Health*. 2023; 23(1): 1575. 37596579 10.1186/s12889-023-16484-z PMC10436410

[16] Hopkins S, Black AA, White S, Wood JM. Visual information processing skills are associated with academic performance in grade 2 school children. *Acta Ophthalmol*. 2019; 97(8): e1141–e1148. 31228337 10.1111/aos.14172

[17] Han X, Ruan X, Zhang Y, et al. Effect of myopia severity on the health-related quality of life in children and their parents. *Curr Eye Res*. 2023; 48(12): 1189–1194. 37655440 10.1080/02713683.2023.2250581

[18] Wang X, Yi H, Lu L, et al. Population prevalence of need for spectacles and spectacle ownership among urban migrant children in eastern China. *JAMA Ophthalmol*. 2015; 133(12): 1399–1406. 26426113 10.1001/jamaophthalmol.2015.3513

[19] Keel S, Müller A, Block S, et al. Keeping an eye on eye care: monitoring progress towards effective coverage. *Lancet Glob Health*. 2021; 9(10): e1460–e1464. 34237266 10.1016/S2214-109X(21)00212-6 PMC8440222

[20] Duan C, Zhou F. A study on the latest status of left-behind children in China. *Popul Res*. 2005; 29(1): 29–36.

[21] Naël V, Moreau G, Monfermé S, et al. Prevalence and associated factors of uncorrected refractive error in older adults in a population-based study in France. *JAMA Ophthalmol*. 2019; 137(1): 3–11. 30326038 10.1001/jamaophthalmol.2018.4229 PMC6439784

[22] Wei S, Sun Y, Li SM, et al. Visual impairment and spectacle use in university students in Central China: the Anyang University Students Eye Study. *Am J Ophthalmol*. 2019; 206: 168–175. 31078530 10.1016/j.ajo.2019.04.026

[23] Ramírez-Ortiz MA, Amato-Almanza M, Romero-Bautista I, et al. A large-scale analysis of refractive errors in students attending public primary schools in Mexico. *Sci Rep*. 2023; 13(1): 13509. 37598286 10.1038/s41598-023-40810-5 PMC10439951

[24] Foreman J, Xie J, Keel S, Taylor HR, Dirani M. Treatment coverage rates for refractive error in the National Eye Health survey. *PLoS One*. 2017; 12(4): e0175353. 28407009 10.1371/journal.pone.0175353 PMC5391052

[25] Malhotra S, Kalaivani M, Rath R, et al. Use of spectacles for distance vision: coverage, unmet needs and barriers in a rural area of North India. *BMC Ophthalmol*. 2019; 19(1): 252. 31830950 10.1186/s12886-019-1262-3 PMC6909564

[26] Wang H, Li Y, Qiu K, et al. Prevalence of myopia and uncorrected myopia among 721 032 schoolchildren in a city-wide vision screening in southern China: the Shantou Myopia Study. *Br J Ophthalmol*. 2023; 107(12): 1798–1805. 36198476 10.1136/bjo-2021-320940

[27] Ma Y, Zhang X, He F, et al. Visual impairment in rural and migrant Chinese school-going children: prevalence, severity, correction and associations. *Br J Ophthalmol*. 2022; 106(2): 275–280. 33127829 10.1136/bjophthalmol-2020-317072

[28] Wang H, Barket B, Du S, et al. The prevalence and correlates of vision impairment and glasses ownership among ethnic minority and Han schoolchildren in rural China. *PLoS One*. 2021; 16(8): e0256565. 34460851 10.1371/journal.pone.0256565 PMC8405009

[29] Wang W, Yan W, Müller A, Keel S, He M. Association of socioeconomics with prevalence of visual impairment and blindness. *JAMA Ophthalmol*. 2017; 135(12): 1295–1302. 29049446 10.1001/jamaophthalmol.2017.3449 PMC6583541

[30] Kearney S, Strang NC, Lewsey J, Azuara-Blanco A, Jonuscheit S. Socio-economic differences in accessing NHS spectacles amongst children with differing refractive errors living in Scotland. *Eye*. 2022; 36(4): 773–780. 33875827 10.1038/s41433-021-01536-8 PMC8956614

[31] Gupta V, Saxena R, Vashist P, et al. Spectacle coverage among urban schoolchildren with refractive error provided subsidized spectacles in North India. *Optom Vis Sci*. 2019; 96(4): 301–308. 30907856 10.1097/OPX.0000000000001356

[32] Jan C, Li SM, Kang MT, et al. Association of visual acuity with educational outcomes: a prospective cohort study. *Br J Ophthalmol*. 2019; 103(11): 1666–1671. 30658989 10.1136/bjophthalmol-2018-313294

[33] Hark LA, Thau A, Nutaitis A, et al. Impact of eyeglasses on academic performance in primary school children. *Can J Ophthalmol*. 2020; 55(1): 52–57. 31712026 10.1016/j.jcjo.2019.07.011

[34] Rajabpour M, Kangari H, Pesudovs K, et al. Refractive error and vision related quality of life. *BMC Ophthalmol*. 2024; 24(1): 83. 38388340 10.1186/s12886-024-03350-8 PMC10885569

[35] Quigley C, Zgaga L, Vartsakis G, Fahy G. Refractive error and vision problems in children: association with increased sedentary behavior and reduced exercise in 9-year-old children in Ireland. *J AAPOS*. 2019; 23(3): 159.e1–159.e6. 10.1016/j.jaapos.2018.12.011 31103561

[36] Zhang J, Wu Y, Sharma B, Gupta R, Jawla S, Bullimore MA. Epidemiology and burden of astigmatism: a systematic literature review. *Optom Vis Sci*. 2023; 100(3): 218–231. 36749017 10.1097/OPX.0000000000001998 PMC10045990

[37] Wang X, Ma Y, Hu M, et al. Teachers’ influence on purchase and wear of children's glasses in rural China: the PRICE study. *Clin Exp Ophthalmol*. 2019; 47(2): 179–186. 30117241 10.1111/ceo.13376

[38] Alemayehu AM, Belete GT, Adimassu NF. Knowledge, attitude and associated factors among primary school teachers regarding refractive error in school children in Gondar city, Northwest Ethiopia. *PLoS One*. 2018; 13(2): e0191199. 29447172 10.1371/journal.pone.0191199 PMC5813908

[39] Evans JR, Morjaria P, Powell C. Vision screening for correctable visual acuity deficits in school-age children and adolescents. *Cochrane Database Syst Rev*. 2018; 2(2): CD005023. 29446439 10.1002/14651858.CD005023.pub3 PMC6491194

[40] Wang X, Congdon N, Ma Y, et al. Cluster-randomized controlled trial of the effects of free glasses on purchase of children's glasses in China: the PRICE (Potentiating Rural Investment in Children's Eyecare) study. *PLoS One*. 2017; 12(11): e0187808. 29161286 10.1371/journal.pone.0187808 PMC5697864

[41] Morjaria P, Evans J, Murali K, Gilbert C. Spectacle wear among children in a school-based program for ready-made vs custom-made spectacles in India: a randomized clinical trial. *JAMA Ophthalmol*. 2017; 135(6): 527–533. 28426857 10.1001/jamaophthalmol.2017.0641 PMC5847082

[42] Ma Y, Congdon N, Shi Y, et al. Effect of a local vision care center on eyeglasses use and school performance in rural China: a cluster randomized clinical trial. *JAMA Ophthalmol*. 2018; 136(7): 731–737. 29801081 10.1001/jamaophthalmol.2018.1329 PMC6136039

[43] Gajiwala UR, Patel RU, Sudhan A, et al. Compliance of spectacle wear among school children. *Indian J Ophthalmol*. 2021; 69(6): 1376–1380. 34011704 10.4103/ijo.IJO_1801_20 PMC8302287

[44] Pawar N, Ravindran M, Renagappa R, Ravilla T, Raman R, Uduman MS. Non-compliance for wearing spectacles: prevalence and determinants in school-going children in South India. *Indian J Ophthalmol*. 2023; 71(2): 608–613. 36727371 10.4103/ijo.IJO_1106_22 PMC10228958

[45] Narayanan A, Kumar S, Ramani KK. Spectacle compliance among adolescents in Southern India: perspectives of service providers. *Indian J Ophthalmol*. 2018; 66(7): 945–949. 29941737 10.4103/ijo.IJO_27_18 PMC6032716

[46] Sabharwal S, Nakayoshi A, Lees CR, Perez S, de Alba CA. Prevalence and factors associated with eyeglass wear compliance among preschoolers from low-income families in San Francisco, California. *JAMA Ophthalmol*. 2021; 139(4): 433–440. 33599687 10.1001/jamaophthalmol.2020.7053 PMC7893547

[47] Lambert SR, Dubois L, Cotsonis G, Hartmann EE, Drews-Botsch C. Spectacle adherence among four-year-old children in the Infant Aphakia Treatment Study. *Am J Ophthalmol*. 2019; 200: 26–33. 30633891 10.1016/j.ajo.2018.12.017 PMC6445735

[48] Wu L, Feng J, Zhang M. Implementing interventions to promote spectacle wearing among children with refractive errors: a systematic review and meta-analysis. *Front Public Health*. 2023; 11: 1053206. 36969641 10.3389/fpubh.2023.1053206 PMC10036364

[49] Yi H, Zhang H, Ma X, et al. Impact of free glasses and a teacher incentive on children's use of eyeglasses: a cluster-randomized controlled trial. *Am J Ophthalmol*. 2015; 160(5): 889–896.e1. 26275472 10.1016/j.ajo.2015.08.006

[50] South J, Roberts P, Gao T, Black J, Collins A. Development of a spectacle wear monitor system: SpecsOn Monitor. *Transl Vis Sci Techn*. 2021; 10(12): 11. 10.1167/tvst.10.12.11 PMC8496409 34614165

[51] Sankaridurg P, Hfe X, Naduvilath T, et al. Comparison of non-cycloplegic and cycloplegic autorefraction in children. *Invest Ophthalmol Vis Sci*. 2017; 58(11): 4451–4461.

