# Supplementary material for: Refractive Error Coverage and Related Sociodemographic Factors: A Study of 1.1 Million Students in Schools of Hubei Province, China
Source: Transl Vis Sci Technol. 2026 Aug 7;15(8):5. doi: 10.1167/tvst.15.8.5 (PMC13456061; doi:10.1167/tvst.15.8.5)
Supplement: Supplementary file 1 [file tvst-15-8-5_s001.pdf]

Supplementary Appendix: Special Questionnaire on Students' Eye Care Habits and Influencing Factors

(For Primary and Junior High School Use)

(Dear student, this questionnaire is designed to investigate the behavioral factors influencing student myopia and related health conditions. It does not collect sensitive personal privacy information. Please complete this form together with your parents or guardians by checking the appropriate numbers/options.)

School___________ Class_________ Name_________ Age________

Gender_________ Ethnicity_________

A. Basic Information and Family Background

	Survey content	Options
A01	How old were your parents when you were born?	1. Father: __ years old 2. Mother: __ years old<br>3. Unknown
A02	Do any family members have myopia? (Multiple choices allowed) *(Mild: < 300 degrees [<-3.00 D]; Moderate: 300-600 degrees [-3.00 D to -6.00 D]; Severe: > 600 degrees [>-6.00 D])*	1. None<br>2. Father has myopia, degree of myopia: _____<br>3. Mother has myopia, degree of myopia: ______<br>4. Other family members have myopia: ______<br>5. Unknown
A03	Father's highest education level:	1. Junior high school or below<br>2. Senior high school (including vocational high school)<br>3. College/University (bachelor's or associate degree)<br>4. Master's degree or above<br>5. Unknown
A04	Mother's highest education level:	1. Junior high school or below<br>2. Senior high school (including vocational high school)<br>3. College/University (bachelor's or associate degree)<br>4. Master's degree or above<br>5. Unknown
A05	Over the past semester, what was your (or your child's) overall academic performance in the class?	1. Excellent (Top 20% of the class)<br>2. Good (21% - 50% of the class, upper middle)<br>3. Average (51% - 80% of the class, lower middle)<br>4. Poor (Bottom 20% of the class)
A06	Do you have biological siblings?	1. No (Only child)<br>2. Yes, I am the ____ child among my siblings

		(Leave blank if only child)
A07	Are you a left-behind child? *(Defined as a child under 18 years of age who remains in their rural hometown while one or both parents migrate to other areas for work for at least 6 months.)*	1. Yes 2. No 3. Unsure

B. Reading, Writing, and Physical Exercise

	调查内容	选项
B01	How much time do you spend using electronic devices (tablets, smartphones, computers, etc.) per day on school days?	1. < 1 hour<br>2. 1～2 hours<br>3. 2～3 hours<br>4. >3 hours
B02	How much time do you spend using electronic devices (tablets, smartphones, computers, etc.) per day on rest days?	1. < 1 hour<br>2. 1～2 hours<br>3. 2～3 hours<br>4.>3 hours
B03	How much time do you spend studying (without using electronic devices) per day on school days?	1. < 1 hour<br>2. 1～2 hours<br>3. 2～3 hours<br>4. 4.>3 hours
B04	How much time do you spend studying (without using electronic devices) per day on rest days?	1. < 1 hour<br>2. 1～2 hours<br>3. 2～3 hours<br>4.>3 hours
B05	How much time do you spend outdoors during the daytime (excluding nighttime) per day on school days?	1. < 1 hour<br>2. 1～2 hours<br>3. 2～3 hours<br>4.>3 hours
B06	How much time do you spend outdoors during the daytime (excluding nighttime) per day on rest days?	1. < 1 hour<br>2. 1～2 hours<br>3. 2～3 hours<br>4.>3 hours

C. Parents' Knowledge of Eye Health (To be answered by parents or guardians)

	调查内容	选项
C01	Do you believe that children diagnosed with "true myopia" can be cured and have their normal vision restored through eye massages, instrumental physiotherapy, or acupuncture?	1. Yes, it can be cured<br>2. No, it cannot be cured<br>3. Unsure
C02	If your child is diagnosed with myopia by an ophthalmologist and prescribed	1.Yes, it will cause faster progression<br>2.No, it will instead help slow down the

	spectacles, do you believe that "wearing appropriate spectacles early" will cause the child's myopia to progress faster?	progression of myopia<br>3. Unsure
C03	Do you believe that "cycloplegic refraction" (using eye drops to relax the ciliary muscle) will cause long-term harm to your child's eyes?	1. Yes 2. No 3. Unsure
C04	Are you aware that high myopia (usually defined as > 600 degrees [> -6.00 D]) not only causes decreased vision but also significantly increases the future risk of retinal detachment, glaucoma, and macular degeneration?	1. Aware<br>2. Unaware<br>3. Completely have no idea
C05	Some parents believe that "it doesn't matter if my child has myopia now; they can be completely cured by laser refractive surgery (e.g., FS-LASIK, SMILE) when they grow up." Are you aware that laser surgery merely changes the corneal thickness to "remove the need for glasses," but cannot reverse the elongation of the axial length caused by myopia or the potential risks of fundus diseases?	1. Aware<br>2. Unaware<br>3. Never paid attention to this
